# Breeding signature of combining ability improvement revealed by a genomic variation map from recurrent selection population in *Brassica napus*

**DOI:** 10.1038/srep29553

**Published:** 2016-07-14

**Authors:** Xinwang Zhao, Bao Li, Ka Zhang, Kaining Hu, Bin Yi, Jing Wen, Chaozhi Ma, Jinxiong Shen, Tingdong Fu, Jinxing Tu

**Affiliations:** 1 Huazhong Agricultural University, National Key Laboratory of Crop Genetic Improvement, National Sub-center of Rapeseed Improvement in Wuhan, Wuhan 430070, China

## Abstract

Combining ability is crucial for parent selection in crop hybrid breeding. The present investigation and results had revealed the underlying genetic factors which might contribute in adequate combining ability, further assisting in enhancing heterosis and stability. Here, we conducted a large-scale analysis of genomic variation in order to define genomic regions affecting the combining ability in recurrent selection population of rapeseed. A population of 175 individuals was genotyped with the Brassica60K SNP chip. 525 hybrids were assembled with three different testers and used to evaluate the general combining ability (GCA) in three environments. By detecting the changes of the genomic variation, we identified 376 potential genome regions, spanning 3.03% of rapeseed genome which provided QTL-level resolution on potentially selected variants. More than 96% of these regions were located in the C subgenome, indicating that C subgenome had sustained stronger selection pressure in the breeding program than the A subgenome. In addition, a high level of linkage disequilibrium in rapeseed genome was detected, suggesting that marker-assisted selection for the population improvement might be easily implemented. This study outlines the evidence for high GCA on a genomic level and provided underlying molecular mechanism for recurrent selection improvement in *B. napus*.

Crop domestication and improvement have enhanced yield, plant habits, and quality. At the genetic level, these phenotypic shifts are the result of a strong selection of targeting genomic regions or genes. Most domesticated plants have experienced a “selection bottleneck” which reduces genetic diversity as compared to their precursor^1^. The reduction in genetic diversity across loci provides insights into the demographic history of domestication. Modern breeding has a similar effect on reducing genetic basis. With the artificial selection of crops, genetic diversity is found to be reduced faster than it was during the original domestication^2^. Effects of these changes have a great potential for breeding, and can facilitate the consistent crop improvement. Selection of the desirable alleles leads to a more drastic loss of genetic variation because individuals carrying favored alleles contributed to each subsequent generation, and those with adverse alleles were gradually eliminated from the population^3^. Identifying the genomic loci is essential for revealing the underlying genetic basis of the traits, and improving breeding efficiency through marker-assisted selection. With the development of sequencing and DNA microarray technology, the genome wide analysis can be used to scan genes, QTLs or genome regions to obtain desirable traits^4^,^5^,^6^,^7^. The frequency of these desirable alleles can increase in the population if they are subjected to selection. Therefore, detecting the allele frequency of the breeding population before and after selection, or comparison with their wild or contrasting population can assist in identifying genes of interest^5^,^8^. For example, alleles of genes that contribute to increased fruit size in tomato^9^, and increased apical dominance in maize^10^, have a high frequency in modern varieties, whereas low frequency in their wild relatives.

Crop breeding programs have generated excellent resources that can be used to improve agronomic traits and identify favorable loci affected by artificial selection. Analysis of genetic diversity, allele frequency, and heterozygosity are used to find genomic alterations and genetic effects on the traits in different generations or sub populations^11^. Additionally, this has been found to be a good approach for scanning genome regions, even candidate genes that underline selection^7^. In chicken, 82 putatively selected regions with reduced levels of heterozygosity are identified^12^. In a cattle population, genetic changes are detected, and 13 genomic regions were found to affect milk production^13^. Moreover, several functional genes were verified in some selected regions in cattle^14^. Similar studies have been carried out in other animals^15^,^16^. In miaze, a set of genes (2~4% of 774 genes) are found to have undergone artificial selection during domestication^3^. Scanning of few known functional genes involved in maize domestication has indicated selection signatures on the genomic level^4^,^17^. Furthermore, several chromosome segments and genes were revealed by comparing genetic variation between wild and cultivated populations in soybean^5^. As for rice, a genealogical history analysis of overlapping low diversity regions can distinguish genomic backgrounds between *indica* and *japonica* rice populations, and 13 additional candidate genes were identified^18^. Another study found 200 genomic regions, spanning 7.8% of the rice genome that had been differentially selected between two putative heterotic groups^19^. These studies have successfully investigated genome-wide genetic changes during domestication and modern breeding. The results can provide useful information to reveal the agronomic potential of a breeding line and genomic loci.

Rapeseed (*Brassica napus*; AACC, 2n = 38) is one of the most important oil crops worldwide. Rapeseed originated from a doubling event between *Brassica rapa* (AA, 2n = 20) and *Brassica oleracea* (CC, 2n = 18) along the Mediterranean coastline 10,000 years ago^20^,^21^. It is considered as a young species because of a short domestication history spanning only 400–500 years^22^. In addition to several other factors, modern breeding has substantially increased production, especially through heterosis. In a hybrid breeding program, combining ability is a crucial factor for parental line selection and for the development of superior hybrids. Evaluation of the combining ability using traditional methods is labor intensive and time-consuming, and may create a bottleneck in hybrid breeding^23^. Therefore, dissection and comparison of the genetic basis of combining ability can be crucial for breeding. Combining ability was defined as a complex trait in plants, and was evaluated by several techniques, including molecular markers, QTL mapping, and genome scan approaches^24^,^25^,^26^. There have been limited investigations carried out to evaluate the genetic basis of combining ability in rapeseed. During rapeseed breeding history, heterosis and double-low varieties (low erucic acid and low glucosinolate) were mainly used to produce higher yield and better quality at the cost of genetic diversity^27^,^28^. Recently, new genetic resources are used to increase the genetic basis of rapeseed, including the artificially synthesized *B. napus* generated from *B. oleracea* and *B. rapa*^29^, the subgenome materials^30^,^31^. Multigenerational improvement and a recurrent selection program are required before utilizing these new materials.

In our work, genomic SNP markers were used to analyze the breeding signatures of GCA as revealed by the genetic variation in a recurrent selection population. The objectives of our study were (1) to estimate genetic diversity of genome-wide SNPs in different groups of the rapeseed restorer population, (2) to detect the putatively selected regions and SNPs associated with breeding efforts on the genomic level, and (3) to identify known important QTLs associated with rapeseed agronomic traits in selected regions. These findings might be of potential use in improving the rapeseed breeding.

## Results

### Phenotype variations in yield and yield-related GCA

Plant yield from the population of 175 families and 525 hybrids, were analyzed with two replicates in three different environments. GCA of each parental line was estimated statistically using the phenotype data sets. Extensive phenotype variations were observed (Table 1). The mean yield of three environments were 13.93 g, 7.41 g, 8.08 g per plant, respectively, and varied from 4.36~36.87 g in Wuhan, from 1.62~15.92 g in Xiangyang and from 2.24~37.82 g in Yichang. The plant yield had high coefficients of variation in the three environments, suggesting that the yield of the rapeseed was a typical quantitative trait and was substantially affected by the environment. The mean value of GCA (Table 1) was 1.34, varied from −3.98~7.98 and the value for coefficient of variation was 172.23%.

### SNP filtering and genetic analysis

After genotyping, out of 52,157 SNPs, a total of 47,986 were called successfully. SNPs with no polymorphism, and missing value > 10% were removed from genotype data sets, and 39,582 SNPs remained. The precise physical location of the SNPs was established by comparative analysis of the reference genome. A total of 27,049 high quality genome-wide SNPs were used for further analysis. These SNPs covered 641.91 Mb of rapeseed genome. The average physical distance between two SNPs was about 30 Kb. Out of 27,049 SNPs, 13,929 were located on the A subgenome, and 13,120 SNPs were on the C subgenome. Statistical analysis of the PIC value (Table 2) revealed that the average PIC value of the A4 chromosome was the highest (0.311), whereas that of the A9 chromosome was the lowest (0.263). Approximately 76.6% of these SNPs have an over medium polymorphism level of 0.25. The polymorphic level of the A subgenome was little higher than that of the C subgenome (Table 2).

### Genetic variation detecting across the regions of the specific loci

**Figure 1:**
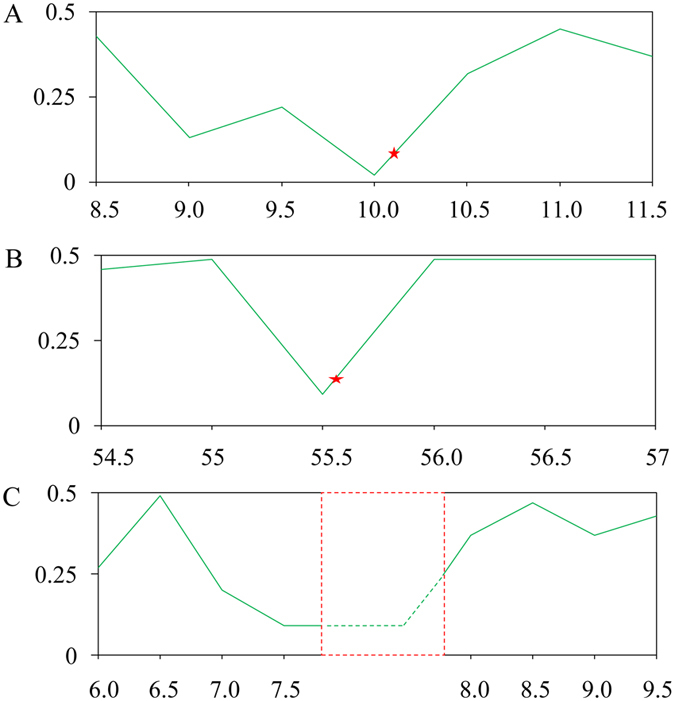
Genetic diversity across the regions of specific loci in the rapeseed genome. (**A**) Genome regions across the erucic acid related gene loci on the chromosomes A8. (**B**) Genome regions across the erucic acid related gene loci on the chromosome C3. (**C**) Genome regions across the Ogura CMS restorer gene *Rf*_*o*_ loci on the chromosome C9. The horizontal axis stands for the physical distance (Mb) of the chromosome, the vertical axis stands for the value of genetic diversity. The red star mark the position of gene located on chromosome; the red dotted box marks the possible inserted position of the gene *Rf*_*o*_.

SNPs were used to detect the genetic variation of three specific loci in rapeseed genome: erucic acid related genes at the *BnFAE1.1, BnFAE1.2* loci on A8 and C3 chromosomes^32^, and the Ogrua CMS restorer gene *Rf*_*o*_ loci on C9 chromosome^33^. It consistently showed that closer the target loci, lower the genetic variation (Fig. 1). These findings indicated that our selection program have been carried out efficiently. Moreover, the evaluation method via genetic diversity could be of potential use in breeding improvement.

### Linkage disequilibrium (LD) in the R population

**Figure 2:**
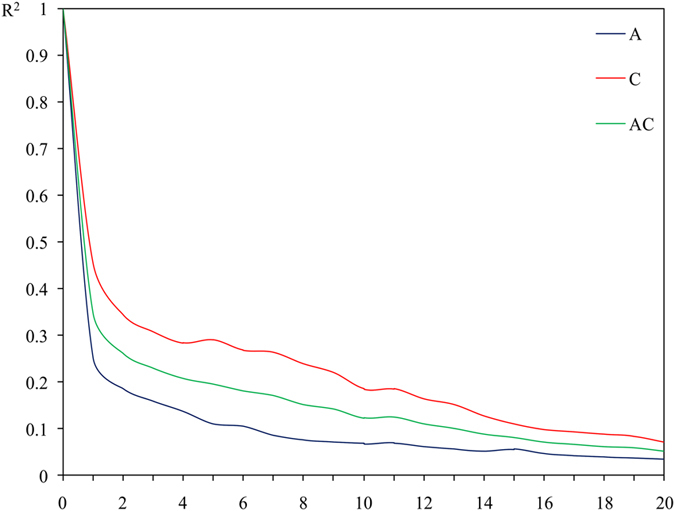
Genome-wide LD decay for the 175 R lines. A, C and AC represent the A and C subgenome, and the whole genome of rapeseed, respectively. The unit distance is 500 Kb.

R^2^ was used to calculate the LD level. For r^2^ = 0.2, LD level occurred at approximately 0.8 Mb, 4.8 Mb, and 2.4 Mb for A and C subgenomes, and AC genome, respectively (Fig. 2). When r^2^ decayed to 0.1, LD values increased to 3 Mb, 8 Mb and 6.5 Mb for A and C subgenomes, and AC genome, respectively. The C subgenome had a larger LD value than A subgenome. As for chromosomes of the two subgenomes, LD of chromosomes in A subgenome was highly consistent, while variation was detected in C subgenome. The LD of the C4 chromosome was higher than 6 Mb when r^2^ = 0.1 (Fig. S2). C1 and C2 chromosomes showed almost no LD decay. Genetic variation of the two subgenomes was also evaluated. The A subgenome had a little higher genetic diversity than the C subgenome (Table 3). By detecting the changes in genetic diversity between the selected and basic populations, we found a greater decrease in the C subgenome (2.7%) as compared to the A subgenome (1.55%).

### Selected regions and candidate QTLs analysis

**Figure 3:**
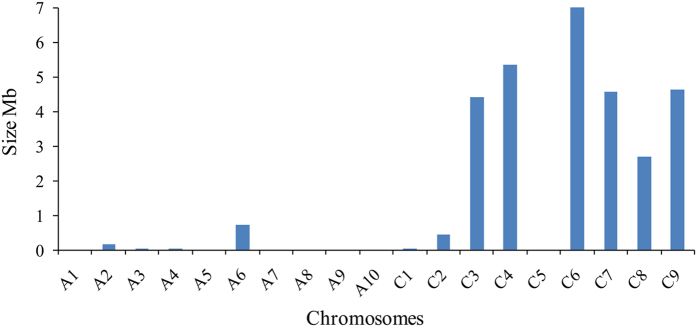
Statistics and distribution of the selected regions in the rapeseed genome. The vertical axis stands for the summary size of selected regions on each chromosome.

**Figure 4:**
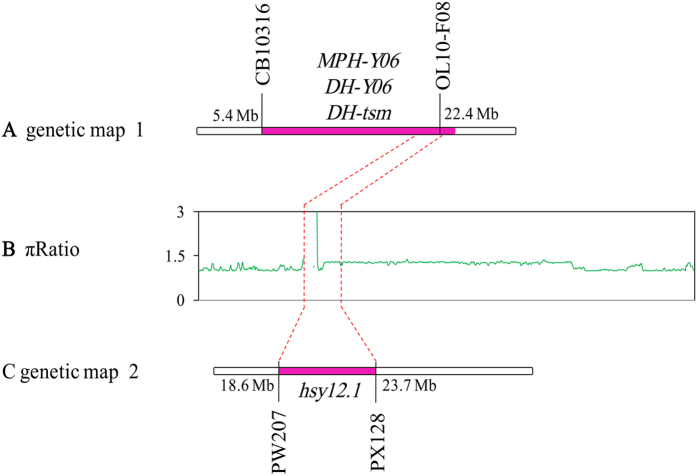
QTLs located in the selected regions on the chromosome C2. (**A**) Genetic map of QTL *MPH-y06, DH-y06, DH-tsm* related to rapeseed yield and seed weight (Basunanda *et al*. 2010). (**B**) Different selection on the chromosome C2 with πRatio. The horizontal axis stands for the chromosome, the vertical axis stands for the πRatio. (**C**) Genetic map of QTL *hsy12.1* related to rapeseed yield (Quijada *et al*. 2006).

Scanning of genomic regions indicated a reduction in genetic diversity. In total, we identified 376 selected regions, covering 3.03% (21.26 Mb) of the assembled genome (Table 4; Fig. S3). More than 96% of these regions were distributed on the C subgenome (Fig. 3; Table 4). C6 chromosome had the largest size of selected regions (4.56 Mb), while A3 had the smallest (0.02 Mb). Furthermore, A1, A5, A7, A8, A9, A10 and C5 chromosomes had no distribution of selected regions. The mean size of selected regions for each chromosome on A and C subgenomes and AC genome were 0.14 Mb, 2.55 Mb, and 1.52 Mb, respectively. The C subgenome had a larger distribution of selected regions than the A subgenome. Many QTLs related to yield and yield-related traits were located in these selected regions (Supplementary Table S1) which likely contributed to the increase in rapeseed yield and GCA. Among the 19 chromosomes of the rapeseed genome, we found differences in the distribution of genetic diversity in the selected regions. In particular, both the chromosomes C2 and C3 had a genome region (C2: 22.0~22.5 Mb; C3: 8.1~8.3 Mb) and distributed throughout the block which lost the genetic variation. For C2 chromosome, we detected the QTL *hsy12.1* and *DHy06*, both previously reported to contribute in rapeseed yield^34^,^35^ (Fig. 4; Supplementary Table S1). Moreover, this region was a QTL hot spot and also contain the QTL of *DH-tsm* for seed weight and the QTL *MPH-y06* for heterosis (Middle Parent Heterosis, MPH)^35^. QTL hot spots contained important QTLs for rapeseed yield and yield-related traits were also detected in the region on chromosome C3 (Supplementary Table S1). All these QTLs in the selected regions provided a potential resource for rapeseed breeding, and selection for these QTLs for rapeseed genetic improvement might lead to low genetic diversity in these regions, but increase in rapeseed yield.

### Pedigree breeding history reproduction

The genomic changes that occurred between the genealogy lines were detected. We reconstructed the recombination events that gave rise to specific inbred lines *zhongshuang5* and *zhongshuang4*, which were both produced from *zhongyou821*. We traced the chromosome segments through pedigree breeding of the two lines. In total, *zhongshuang5* inherited 15.41% of its genome from the ancestral line *zhongyou821* while *zhongshuang4* inherited 34.06% (Table 4; Fig. S4). *Zhongshuang5* inherited 24.17% of the A subgenome and 10.26% of the C subgenome from *zhongyou821*, while *zhongshuang4* inherited only 14.37% of the A subgenome but 45.63% of the C subgenome from *zhongyou821*. Out of the 19 chromosomes, six chromosomes (A1, A5, C1, C6, C7 and C8), showed that more than half of their chromosome fragments were inherited from *zhongyou821* into *zhongshuang4*, particularly in C6 and C7, where almost the whole chromosomes were found to be inherited. However, 8 chromosomes (A2, A3, A4, A6, A7, A8, A9, and C2) were not inherited into *zhongshuang4*. In 45.63% inherited component, we observed that 84.39% was from the C subgenome. These findings were consistent with the analysis of the selected regions.

### Fixed SNP provided a reference index for population improvement

**Figure 5:**
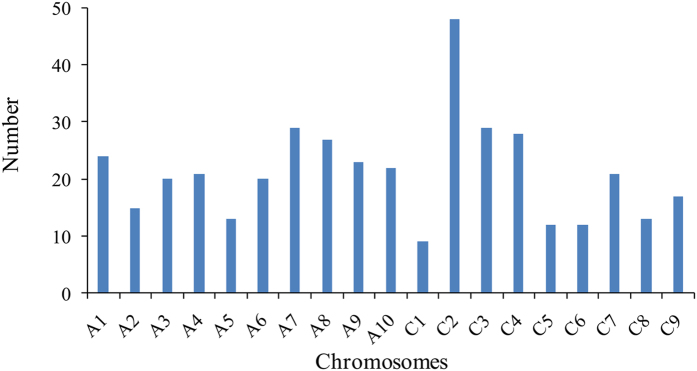
Distribution of the Fixed SNP in the rapeseed genome.

By detecting the allele frequencies of genome-wide SNPs, we identified a total of 403 Fixed SNPs from the genotype data sets. There were 214 of these Fixed SNPs from the A subgenome and 189 from the C subgenome (Fig. 5). The allele frequencies of these SNPs were fixed to 0 or 1 in the selected group and subsequent generations. These loci have lost other alleles and showed monomorphism in the subsequent population.

## Discussion

A yield-improving plateau occurs for a limited genetic diversity^2^. Demonstration and breeding program reduce the crop genetic diversity significantly^1^,^2^. To enhance the diversity, we used the contents of the subgenomes from the relation species in *Brassica*. By the breeding method of recurrent selection for GCA improvement, some desirable loci were maintained in the population and others undesirable loci were deleted from the population. Our analysis provided useful information to exhibit genetic base of GCA on rapeseed.

The LD value of this population was larger than the natural population reported previously. Breeding selection for the favorable alleles would increase the LD level between loci in genome^36^. In this study, we observed strong LD between SNPs separated up to 2.5 Mb (r^2^ = 0.2). This value was higher than the LD value obtained in previous studies^37^,^38^,^39^, which have indicated LD levels at about 500 Kb, 700 Kb, and 2 cM, respectively. In these studies, researchers have used the resource populations collected from all over the world which contained higher rapeseed genetic variation. Contrastingly, in our study, the population was derived from several artificially synthesized *B. napus* and the subgenome materials. Afterwards, it was improved for subsequent generations which might have contributed to the higher LD. These findings might be useful for marker-assisted recurrent selection. Our results also demonstrated higher LD in the C subgenome, especially for C1, C2 and C4 chromosomes, which was consistent with the previous results^40^,^41^. Possibly, this could be explained by several reasons: the C subgenome had a lower level of genetic variability than the A subgenome, and the C subgenome might be under a more intense selection pressure in our breeding program. Polygenetic analysis also showed a decreasing trend in the diversity of the C subgenome than that of the A subgenome (Table 3). It had also provided a favorable evidence for the higher LD of the C subgenome.

The C subgenome is a repository for a wider range of selected regions with favorable loci contributing to rapeseed agronomic traits. By detecting the changes in genomic diversity, we identified 376 genomic regions and covered 3.03% (21.26 Mb) of the rapeseed genome. Many important QTLs related to yield and yield-related traits were located in some of these selected regions (Supplementary Table S1). In particular, some of these genomic regions harboured QTL hotspots (for one trait or multiple traits) or significant QTL reported in other studies (Fig. 4). We noticed that more than 96.05% of these selected regions distributed on the C subgenome (Fig. 3; Table 4) and only about 3.95% was distributed on the A subgenome. This indicated that the C subgenome had sustained more pressure in the selection program or the C subgenome contributed more to the yield-related GCA than the A subgenome. The differences in the genome background between the genealogical lines further support this conclusion (Table 4; Fig. S4). In China, for improvement of the adaptive traits of the European and Japanese varieties, breeders have lead to the introgression of the A genome components of *B. rapa* into the *B. napus* genome^31^,^42^. This process has enhanced the genetic diversity of A subgenome in Chinese rapeseed. However, the breeding potential of C subgenome has not been developed and utilized much. The genetic background of our population contained European winter-type rapeseed, which has higher genetic variation of C subgenome than the A subgenome^37^. Furthermore, the subgenome materials (A^r^A^r^C^c^C^c^) have been introgressed with the C^c^ genome from *B. carinata*, and artificial synthetic materials have been introgressed with the C^o^ genome from *B. oleracea*, which might also contributed to the increased genetic variation of C subgenome in *B. napus*. These new genetic components of the C subgenome might potentially improve the rapeseed yield. Results of the present investigation, along with a deeper understanding of heterosis and changes in breeding programs have indicated that the C subgenome needs to be fully developed in rapeseed hybrid breeding.

Recurrent selection has been established as a very useful method for plant breeding^43^,^44^. The process can break the linkage of disadvantageous alleles and pyramid favorable alleles through sustaining recombination and selection. In this study, we used the recurrent selection method to improve the GCA level of the R population. The top 20% individuals with high GCA were selected for the next generation. Genetic analyses showed that there were many genomic regions under selection. These regions might play an important role in rapeseed breeding. We suggest that most favorable alleles might be accumulated through MAS and standing selection. This might assist in the development and improvement of potential rapeseed.

In summary, we have conducted a comprehensive analysis of changes in genomic variation and identified a number of genomic regions and loci subjected to selection. Firstly, we found a slightly higher level of genetic diversity for the A subgenome as compared to the C subgenome. Both of the subgenomes had a higher LD, and might be beneficial for MAS. Secondly, the program for breeding selection might decrease the genetic diversity of the population and some allelic variations would disappear or approach to fixation. Thirdly, most of the selected regions were distributed on the C subgenome, which indicated that the C subgenome might have been under stronger selection pressure, or contributed more towards GCA improvement in rapeseed hybrid breeding. Finally, we have identified several potential selection targets, genomic regions and loci, which provided further insight into rapeseed research and improvement.

## Materials and Methods

### Plant materials, phenotype evaluation, and GCA estimation

We used two new types of rapeseed (artificial synthetic *B. napus* and subgenomic materials) and winter type rapeseed in the present investigation: (1) 41 artificial synthetic *B. napus* from the University of Goettingen, Germany, were crossed with three winter type lines (*SW0736, SW0740* and *SW0784*) from Sweden in 1999; the F_5_ families were crossed with Ogura-INRA CMS lines and restorer line *R2000* in 2004. (2) Seven subgenomic materials (A^r^A^r^C^c^C^c^)^45^, 2 Pol CMS restorers (*5148R* and *6178R*) and yellow seed coat variety *No2127* were crossed with *R2000*. F_1_ obtained from (1) and (2) was used to construct the recurrent selection population in 2005 in Huazhong Agricultural University, China. A recurrent selection program was used to improve the GCA level of the population. The previous populations were randomly pollinated in an isolated environment. The sterile plants were harvested and the seeds were used to construct the next generation population. Meanwhile, seed quality (oil content, glucosinolate and erucic acid) was considered as an important breeding goal. In 2012, we randomly selected 175 plants (with the *Rf*_*o*_ gene) and crossed them with three different testers (*Yu7-120, Yu7-126* and *Yu7-140*) to produce hybrid seeds following NC II design^46^, resulting in a total of 525 hybrids. All hybrids, and 178 parental lines, were sown in three semi-winter rapeseed environments (Wuhan, 29°58′N, 113° 53′E; Xiangyang, 32° 04′N, 112° 05′E and Yichang, 30′40′N, 111° 45′E) in China. Field trials were followed as completely random design with two replications at each location.

General combining ability (GCA) of each parental lines was calculated using the formula: gi = yi-ŷ, where gi stands for GCA of parental line, yi and ŷ each stand for the mean of crosses with same parent Pi and the mean of all crosses, respectively^47^. Based on the GCA, we set 20% as the selection intensity, which could not rapidly decrease the genetic diversity of the population. Afterwards, 35 lines with a high GCA were selected from the population, defined as the selected population or group.

The other three genealogical lines (*zhongyou821, zhongshuang4*, and *zhongshuang5*, Fig. S1) were used to detect the genome changes in pedigree breeding. The *zhongyou821* is highly considered for rapeseed breeding in China. Many elite inbred lines including both open pollination cultivars and hybrid parents, were developed from this line. For example, both *zhongshuang4* and *zhongshuang5* are derived from *zhongyou821*, and bred as open pollination cultivars. Recently, F_1_ hybid of *zhongshuang4* and Pol CMS lines is found to exhibit excellent heterosis performance. Therefore, *zhongshuang4* is considered as a good restorer line and used to develop several other hybrid cultivars and restorer lines.

### SNP filtering and genotype analysis

Genomic DNA was extracted from young leaves using the cetyl triethyl ammnonium bromide (CTAB) method^48^. The Illumina BrassicaSNP60 Bead Chip containing 52,157 SNPs was employed to genotype this panel of rapeseed. The experiment followed the manufacturer’s protocol as described by Illumina Company. (http://www.illumina.com/technology/infinium_hd_assay.ilmn). The SNP data was clustered and called automatically using the Illumina GenomeStudio genotyping software. SNPs with no polymorphism and missing value > 10% were excluded. The source sequences of the remaining SNPs were identified through BlastN searches against the reference genome sequence of Darmor-*bzh*^49^ (http://www.genoscope.cns.fr/brassicanapus). SNPs with an ambiguous physical position or multiple blast-hits were also excluded from the genotype data sets.

### Polygenetic and linkage disequilibrium analysis

Genetic diversity (π), polymorphism information content (PIC) and alleles frequencies of each SNP on 19 chromosomes were estimated by the PowerMarker software^50^. Linkage disequilibrium (LD) between SNPs was calculated by all markers using the TASSEL software version 5.1^51^. LD decay was evaluated on the basis of the r^2^ value and corresponding distance between two SNPs.

### Selected regions, Fixed SNP and candidate QTL detecting

To calculate diversity changes across the genome, a sliding window method was used to analyze each chromosome separately, with a window size of five SNPs and a sliding step of two SNPs. Ratio of the genetic diversity value of each window between selected and basic populations was used to identify genomic regions affected by selection, which was estimated by the formula: π_Ratio_ = π_basic_/π_selected_. We selected the top 5% windows as candidate regions for further analysis. In addition, we analyzed many reported QTLs of rapeseed yield and yield- related traits. If the closely linked markers or the mapped interval were located in or overlapped with selected regions, we considered them to be candidate selected QTLs. We also calculated the allele frequencies of each SNP on the 19 chromosomes, and identified the SNPs which allele frequencies were changed to a hundred percent in the selected population and defined such SNPs as Fixed SNP.

### Genome changes detecting during the pedigree breeding

We used the Beagle4.1 software^52^ to detect the chromosome segments of identity by descent (IBD)^53^ between the two half-sib sister lines (*zhuangshuang4* and *zhongshuang5*) and their common ancestor (*zhongyou821*) by genome-wide SNP markers. The P value of the significant level was set as 1 × 10^−7^. Uncertain regions (not defined IBD segments) were equally appropriate into the two adjacent blocks. We surveyed the inherited proportion of their genome from *zhongyou821* and set different colors for chromosome segments according to the type of IBD.

## Additional Information

**How to cite this article**: Zhao, X. *et al*. Breeding signature of combining ability improvement revealed by a genomic variation map from recurrent selection population in *Brassica napus. Sci. Rep.* **6**, 29553; doi: 10.1038/srep29553 (2016).

## Supplementary Material

Supplementary Information

## Figures and Tables

**Table 1:** Phenotype variations of plant yield and yield-related GCA.

Trait	Env^a^	Mean Â ± SE^b^	Range	Variance	CV %^c^
Yield	WH	13.93 ± 0.43	4.36~36.87	31.74	40.46%
	XY	7.41 ± 0.22	1.62~15.92	8.07	38.32%
	YC	8.08 ± 0.53	2.24~37.82	48.55	86.23%
GCA^d^	–	1.34 ± 0.17	−3.98~7.98	5.35	172.23%

^a^Environment, WH stands for Wuhan; XY stands for Xiangyang; YC stands for Yichang.

^b^SE is an abbreviation of standard error.

^c^CV is an abbreviation of coefficient of variation.

^d^GCA is an abbreviation of general combining ability.

**Table 2:** Summary of PIC value of rapeseed genome.

Chr	Number of SNPs	PIC^a^ Value							
		<0.1	0.1~0.15	0.15~0.2	0.2~0.25	0.25~0.3	0.3~0.35	0.35~0.4	Ave^b^
A1	1,356	104	62	106	133	196	314	441	0.280
A2	1,046	68	34	55	90	129	276	394	0.295
A3	2,006	96	58	133	181	245	503	790	0.298
A4	1,316	51	54	60	82	120	276	673	0.311
A5	1,440	58	39	64	128	200	345	606	0.305
A6	1,375	67	62	91	111	193	354	497	0.293
A7	1,642	109	74	72	176	205	376	630	0.291
A8	1,043	102	46	35	71	95	227	467	0.293
A9	1,422	115	160	87	133	246	376	305	0.263
A10	1,283	83	50	74	97	146	279	554	0.297
C1	1,803	56	65	38	141	199	854	450	0.300
C2	1,613	65	20	16	49	623	270	570	0.307
C3	2,013	193	113	99	160	258	411	779	0.284
C4	2,350	99	168	61	92	184	1,244	502	0.299
C5	671	68	37	57	58	56	172	223	0.275
C6	1,058	87	33	69	82	96	255	436	0.294
C7	1,467	92	58	84	200	374	276	383	0.277
C8	1,289	86	60	54	75	155	219	640	0.303
C9	856	53	93	30	45	101	186	348	0.288
A	13,929	853(6.1%)	639(4.6%)	777(5.6%)	1,202(8.6%)	1,775(12.7%)	3,326(23.9%)	5,357(38.5%)	0.293
C	13,120	799(6.1%)	647(4.9%)	508(3.9%)	902(6.9%)	2,046(15.6%)	3,887(29.6%)	4,331(33.0%)	0.292
AC	27,049	1,652(6.1%)	1,286(4.8%)	1,285(4.8%)	2,104(7.8%)	3,821(14.1%)	7,213(26.7%)	9,688(35.8%)	0.292

Chr represents the chromosome; A, C and AC represent the A and C subgenome, and the whole genome of rapeseed, respectively.

^a^PIC is an abbreviation of Polymorphism Information Content.

^b^The average PIC value of all the SNPs on the chromosome.

**Table 3:** Genetic diversity of the genome in R population and the selected population.

Chr	π-R^a^	π-S^b^	DR^c^
A	0.370	0.364	1.55%
C	0.368	0.358	2.70%
AC	0.369	0.361	2.10%

Chr represent the chromosome; A, C and AC represent the A and C subgenome, and the whole genome of rapeseed, respectively.

^a^The average value of genetic diversity (π) for the R population.

^b^The average value of genetic diversity (π) for the the selected population.

^c^The decrease ratio of genetic diversity.

**Table 4:** Summary of size and distribution of selected regions and IBD regions between the genealogy lines.

Chr	Genome/bp^a^	SR/bp^b^	IBD (*zy821*-*zs5*)/bp^c^	IBD (*zy821*-*zs4*)/bp^d^	P-*zs5*^e^	P-*zs4*^f^
A1	23,211,631	–	–	18,109,972	–	78.02%
A2	24,779,191	150,550	–	–	–	–
A3	29,440,021	21,159	3,627,709	–	12.32%	–
A4	19,079,334	24,096	–	–	–	–
A5	22,947,632	–	632,434	13,243,517	2.76%	57.71%
A6	24,371,683	714,642	8,289,655	–	34.01%	–
A7	23,866,120	–	8,611,978	–	36.08%	–
A8	18,721,663	–	11,120,209	–	59.40%	–
A9	33,804,806	–	17,411,101	–	51.50%	–
A10	17,337,919	–	7,729,870	2,779,229	44.58%	16.03%
C1	38,753,021	30,525	25,509,072	24,091,334	65.82%	62.17%
C2	46,048,588	448,296	–	–	–	–
C3	60,554,402	4,411,490	10,834,670	29,809,163	17.89%	49.23%
C4	48,651,934	5,365,855	3,813,411	22,449,981	7.84%	46.14%
C5	42,591,603	–	–	1,114,188	–	2.62%
C6	37,161,013	6,995,577	1,343,092	33,985,352	3.61%	91.45%
C7	44,078,365	4,567,179	–	43,849,181	–	99.48%
C8	38,310,205	2,681,712	–	20,778,723	–	54.24%
C9	48,203,342	4,618,106	–	8,442,909	–	17.52%
Sum A	237,560,000	910,447	57,422,956	34,132,718	24.17% (58.05%)	14.37% (15.61%)
Sum C	404,352,473	29,118,740	41,500,245	184,520,831	10.26% (41.95%)	45.63% (84.39%)
Sum AC	641,912,473	30,029,187	98,923,201	218,653,549	15.41%	34.06%

Chr represents the chromosome; A, C and AC represent the A and C subgenomes, and the whole genome of rapeseed, respectively. *Zy821* stands for *zhongyou821*; *zs5* stands for *zhongshuang5*; *zs4* stands for *zhongshuang4*.

^a^Genome size covered by all SNPs on each chromosomes.

^b^Summary size of selected regions on each chromosomes. SR stands for selected region.

^c^Summary size of IBD regions on each chromosomes between *zy821* and *zs5*. IBD is an abbreviation of identity by descent.

^d^Summary size of IBD regions on each chromosomes between *zy821* and *zs4*.

^e^The percentage of the IBD regions shared the chromosome between *zy821* and *zs5*.

^f^The percentage of the IBD regions shared the chromosome between *zy821* and *zs4*.
