# Supplementary material for: Breeding signature of combining ability improvement revealed by a genomic variation map from recurrent selection population in Brassica napus
Source: Sci Rep. 2016 Jul 14;6:29553. doi: 10.1038/srep29553 (PMC4944167; doi:10.1038/srep29553)
Supplement: Supplementary Information [file srep29553-s1.doc]

**Supplementary Information**

**Breeding signature of combining ability improvement revealed by a genomic variation map from recurrent selection population in *Brassica napus***

Xinwang Zhao^1^, Bao Li^1^, Ka Zhang^1^, Kaining Hu^1^, Bin Yi^1^, Jing Wen^1^, Chaozhi Ma^1^, Jinxiong Shen^1^, Tingdong Fu^1^, Jinxing Tu^1*^

^1^ National Key Laboratory of Crop Genetic Improvement, National Sub-center of Rapeseed Improvement in Wuhan, Huazhong Agricultural University, Wuhan 430070, China

* Corresponding author: Jinxing Tu.

Tel: +86-27-87281819;

Fax: +86-27-87280009;

E-mail: [tujx@mail.hzau.edu.cn](mailto:tujx@mail.hzau.edu.cn).

**Figure S1.** Genetic background, pedigree breeding, and cultivate history of three genealogical lines *zhongyou821*, *zhongshuang4*, and *zhongshuang5*.

**Figure S2.** LD decay of the 19 rapeseed chromosomes in the R population.

**Figure S3.** Different selection on rapeseed 19 chromosomes with πRatio.

**Figure S4.** Reconstructed recombination events in *zhongshuang4* and *zhongshuang5* as they were derived from the same parental line *zhongyou821*. Left column stands for the genomic components of *zhongshuang5*, right column stands for the genomic components of *zhongshuang4*.

**Table S1.** Summary of QTLs in selected regions

| QTL | Trait | Chr | Start Pos | End Pos | Key References |
| --- | --- | --- | --- | --- | --- |
| *qSW.A4-1^*^* | Seed weight | A4 | 1,104,975 | 1,203,922 | Shi et al.2011 |
| *DH-y06* | Yield | A6 | 1,865,858 | 1,936,778 | Basunanda et al.2010 |
| *MPH-y06* | Yield | A6 | 1,866,424 | 2,152,572 | Basunanda et al.2010 |
| *MPH-y07* | Yield | A6 | 1,866,424 | 2,152,572 | Basunanda et al.2010 |
| *hph11.3* | Plant height | C1 | 16,875,212 | 16,905,737 | Udall et al. 2006 |
| *sl11* | Silique length | C1 | 16,905,259 | 16,956,909 | Chen et al. 2007 |
| *sdl11* | Silique Density | C1 | 16,905,259 | 16,956,909 | Chen et al. 2007 |
| *qLR10-1* | Resistance to Sclerotinia | C1 | 16,905,259 | 16956909 | Mei et al.2014 |
| *hph12.1* | Plant height | C2 | 22,559,827 | 22,565,597 | Udall et al. 2006 |
| *ph12b* | Plant height | C2 | 22,559,827 | 22,565,597 | Quijada et al. 2006 |
| *hsy2.1* | Yield | C2 | 22,559,827 | 22,565,597 | Quijada et al. 2006 |
| *hdtf12b* | Flowering time | C2 | 22,559,827 | 22,565,597 | Quijada et al. 2006 |
| *DH-y06* | Yield | C2 | 22,559,827 | 22,565,597 | Basunanda et al. 2010 |
| *DH-tsm* | Seed weight | C2 | 22,559,827 | 22,565,597 | Basunanda et al. 2010 |
| *MPH-y06* | Yield | C2 | 22,559,827 | 22,565,597 | Basunanda et al. 2010 |
| *qSY.C3-1* | Yield | C3 | 7,697,498 | 11,125,931 | Shi et al.2011 |
| *qPH.C3-1* | Plant height | C3 | 7,697,498 | 11,125,931 | Shi et al.2011 |
| *qPN.C3-1* | Plant height | C3 | 7,697,498 | 11,125,931 | Shi et al.2011 |
| *qOIL.C3-1* | Oil content | C3 | 7,697,498 | 11,125,931 | Shi et al.2011 |
| *qBY.C3-1* | Biomass yield | C3 | 7,697,498 | 11,125,931 | Shi et al.2011 |
| *qSW.C3-1* | Seed weight | C3 | 7,697,498 | 11,125,931 | Shi et al.2011 |
| *qMT.C3-1* | Flowering time | C3 | 7,697,498 | 11,125,931 | Shi et al.2011 |
| *qHI.C3-1* | Harvest index | C3 | 7,697,498 | 11,125,931 | Shi et al.2011 |
| *hdtf13.1* | Flowering time | C3 | 7,697,498 | 11,125,931 | Quijada et al. 2006 |
| *sw13.1* | Seed weight | C3 | 7,697,498 | 11,125,931 | Quijada et al. 2006 |
| *hsy13.1* | Yield | C3 | 7,697,498 | 11,125,931 | Udall et al. 2006 |
| *hsy13.4* | Yield | C3 | 19,713,742 | 19,722,903 | Udall et al. 2006 |
| *lmi13* | Length of maim inflorescence | C3 | 48,779,239 | 48,820,306 | Chen et al. 2007 |
| *sy13.5* | Yield | C3 | 48,818,895 | 49,528,253 | Udall et al. 2006 |
| *hsw13.3* | Yield | C3 | 50,694,459 | 50,747,800 | Udall et al. 2006 |
| *hsy13.3* | Yield | C3 | 50,829,418 | 51,025,057 | Udall et al. 2006 |
| *sw14.3* | Seed weight | C4 | 3,245,287 | 3,305,387 | Udall et al. 2006 |
| *DH-hct* | Flowering time | C4 | 3,245,287 | 3,305,387 | Basunanda et al. 2010 |
| *bk14a.5* | Beak length | C4 | 5,942,484 | 5,973,245 | Udall et al. 2006 |
| *ph14* | Plant height | C4 | 12,118,302 | 12,191,889 | Chen et al. 2007 |
| *hph14* | Plant height | C4 | 12,118,302 | 12,191,889 | Chen et al. 2007 |
| *fb14* | Primary branch | C4 | 12,118,302 | 12,191,889 | Chen et al. 2007 |
| *TH-stw* | Seed weight | C4 | 31,174,768 | 31,823,206 | Basunanda et al. 2010 |
| *TH-sdw* | Yield | C4 | 31,174,768 | 31,823,206 | Basunanda et al. 2010 |
| *THy06* | Yield | C4 | 34,439,531 | 37,429,357 | Basunanda et al. 2010 |
| *blb16.1* | Bacterial leaf blight | C6 | 2,600,300 | 2,652,345 | Quijada et al. 2006 |
| *hdtf16.2* | Flowering time | C6 | 4,408,059 | 4,823,206 | Quijada et al. 2006 |
| *DH-y05* | Yield | C6 | 4,408,059 | 4,823,206 | Basunanda et al. 2010 |
| *ph16.5* | Plant height | C6 | 12,387,496 | 13,581,326 | Udall et al. 2006 |
| *htw16.4* | Seed weight | C6 | 12,387,496 | 13,581,326 | Udall et al. 2006 |
| *TH-ph06* | Plant height | C6 | 15,765,843 | 15,815,415 | Basunanda et al. 2010 |
| *ph16.3* | Plant height | C6 | 18,223,157 | 18,272,792 | Udall et al. 2006 |
| *bk16a.5* | Beak length | C6 | 18,251,064 | 18,274,607 | Udall et al. 2006 |
| *MPH-tsm07* | Heterosis | C6 | 18,274,607 | 18,959,602 | Basunanda et al. 2010 |
| *TH-tsm05* | Seed weight | C6 | 20,425,584 | 21,099,589 | Basunanda et al. 2010 |
| *blb16.3* | Bacterial leaf blight | C6 | 26,266,327 | 26,337,890 | Udall et al. 2006 |
| *sy16.1* | Yield | C6 | 26,266,327 | 26,337,890 | Quijada et al. 2006 |
| *blb16.1* | Bacterial leaf blight | C6 | 26,302,935 | 26,464,186 | Udall et al. 2006 |
| *sy16.3* | Yield | C6 | 26,302,935 | 26,464,186 | Quijada et al. 2006 |
| *sy16.3* | Yield | C6 | 26,337,890 | 26,473,932 | Udall et al. 2006 |
| *tw16.1* | Seed weight | C6 | 26,337,890 | 26,473,932 | Quijada et al. 2006 |
| *htw16.4* | Seed weight | C6 | 26,464,186 | 27,057,115 | Udall et al. 2006 |
| *hdtf16.3* | Flowering time | C6 | 27,057,115 | 27,060,163 | Udall et al. 2006 |
| *hsy16.3* | Yield | C6 | 27,059,884 | 27,150,896 | Quijada et al. 2006 |
| *ph16.1* | Plant height | C6 | 29,440,197 | 29,525,424 | Quijada et al. 2006 |
| *htw16.4* | Seed weight | C6 | 29,453,535 | 29,572,018 | Quijada et al. 2006 |
| *hl16.3* | Plant height | C6 | 29,525,424 | 29,653,680 | Quijada et al. 2006 |
| *SRC6-1* | Resistance to Sclerotinia | C6 | 32,374,557 | 35,755,536 | Wu et al.2013 |
| *DH-stw* | Seed weight | C7 | 31,157,190 | 32,551,553 | Basunanda et al. 2010 |
| *tw17.1* | Seed weight | C7 | 32,365,089 | 32,521,704 | Quijada et al. 2006 |
| *qHI.C7-1* | Harvest index | C7 | 33,074,937 | 34,814,057 | Shi et al.2011 |
| *sw17.1* | Seed weight | C7 | 39,642,616 | 40,480,139 | Udall et al. 2006 |
| *tw17.1* | Seed weight | C7 | 39,642,616 | 40,480,139 | Udall et al. 2006 |
| *hsy17.3* | Yield | C7 | 39,642,616 | 40,480,139 | Quijada et al. 2006 |
| *tw18.5* | Seed weight | C8 | 13,437,859 | 13,616,160 | Udall et al. 2006 |
| *sw18.5* | Seed weight | C8 | 13,437,859 | 13,616,160 | Udall et al. 2006 |
| *hsy19.3* | Yield | C9 | 41,091,545 | 41,122,578 | Quijada et al. 2006 |
| *qPY.C9-3* | Yield | C9 | 41,091,545 | 41,122,578 | Shi et al.2011 |
| *ld19.3* | Lodging | C9 | 41,095,825 | 41,121,645 | Udall et al. 2006 |
| *ld19.5* | Length of maim inflorescence | C9 | 41,121,333 | 41,122,578 | Udall et al. 2006 |

**References:**

Basunanda, P., Radoev, M., Ecke, W., Friedt, W., Becker, H. C., & Snowdon, R. J. (2010). Comparative mapping of quantitative trait loci involved in heterosis for seedling and yield traits in oilseed rape (Brassica napus L.). *Theoretical and Applied Genetics*, *120*(2), 271-281.

| Chen, W., Zhang, Y., Liu, X., Chen, B., Tu, J., & Tingdong, F. (2007). Detection of QTL for six yield-related traits in oilseed rape (Brassica napus) using DH and immortalized F2 populations. *Theoretical and Applied Genetics*, *115*(6), 849-858. |
| --- |
| Mei, J., Ding, Y., Lu, K., Wei, D., Liu, Y., Disi, J. O., ... & Qian, W. (2013). Identification of genomic regions involved in resistance against Sclerotinia sclerotiorum from wild Brassica oleracea. Theoretical and applied genetics,126(2), 549-556. |
| Quijada, P. A., Udall, J. A., Lambert, B., & Osborn, T. C. (2006). Quantitative trait analysis of seed yield and other complex traits in hybrid spring rapeseed (Brassica napus L.): 1. Identification of genomic regions from winter germplasm. *Theoretical and Applied Genetics*, *113*(3), 549-561. |
| Shi, J., Li, R., Zou, J., Long, Y., & Meng, J. (2011). A dynamic and complex network regulates the heterosis of yield-correlated traits in rapeseed (Brassica napus L.). *PloS one*, *6*(7), 371. |
| Udall, J. A., Quijada, P. A., Lambert, B., & Osborn, T. C. (2006). Quantitative trait analysis of seed yield and other complex traits in hybrid spring rapeseed (Brassica napus L.): 2. Identification of alleles from unadapted germplasm. *Theoretical and Applied Genetics*, *113*(4), 597-609. |

Wu, J., Cai, G., Tu, J., Li, L., Liu, S., Luo, X., ... & Zhou, Y. (2013). Identification of QTLs for resistance to Sclerotinia stem rot and BnaC. IGMT5. a as a candidate gene of the major resistant QTL SRC6 in Brassica napus. *PloS one*, *8*, e67740.
